# Durable complete response for oligometastatic renal cell carcinoma with immune checkpoint inhibition and cytoreductive nephrectomy in a Jehovah’s witness: A case report

**DOI:** 10.3389/fonc.2022.949400

**Published:** 2022-12-02

**Authors:** Tony Zibo Zhuang, Lara Harik, Seth Force, Agreen Hadadi, Mehmet Asim Bilen, Jacqueline T. Brown, Bradley C. Carthon, Jamie Goldman, Omer Kucuk, Viraj A. Master, Bassel Nazha

**Affiliations:** ^1^ School of Medicine, Emory University, Atlanta, GA, United States; ^2^ Department of Pathology and Laboratory Medicine, Emory University Hospital, Atlanta, GA, United States; ^3^ Winship Cancer Institute of Emory University, Atlanta, GA, United States; ^4^ Division of Cardiothoracic Surgery, Department of Surgery, School of Medicine, Emory University, Atlanta, GA, United States; ^5^ Department of Hematology and Medical Oncology, School of Medicine, Emory University, Atlanta, GA, United States; ^6^ Department of Urology, School of Medicine, Emory University, Atlanta, GA, United States

**Keywords:** renal cell carcinoma, nivolumab, ipilimumab, cytoreductive, oligometastatic, TKI (tyrosine kinase inhibitor)

## Abstract

The role of cytoreductive nephrectomy in patients with metastatic renal cell carcinoma is a subject of debate. We report a durable complete response in a 62-year-old man Jehovah’s Witness with metastatic clear cell renal cell carcinoma who received two cycles of nivolumab/ipilimumab followed by radical nephrectomy and metastasectomy of known pulmonary disease site, both without a clinical need for perioperative blood transfusions. The patient continues to be without evidence of disease and without additional need for systemic therapy over a year after his radical nephrectomy. The case highlights that cytoreductive nephrectomy continues to play a role in the era of immune checkpoint inhibitors.

## Introduction

Renal cancer is the eighth most common cause of cancer worldwide, responsible for over 13,000 deaths annually (1). Clear cell renal cell carcinoma (ccRCC) is the dominant histological group, accounting around 75% of RCC cases (2). Upfront surgical resection is the gold standard treatment in patients with non-metastatic disease. Around 16% of renal cancers involve metastases at initial diagnosis with a 5-year survival rate of approximately 14%. The IMDC (International Metastatic RCC Database Consortium) predicts survival in patients with metastatic renal cell carcinoma (mRCC) and was originally validated in patients receiving vascular endothelial growth factor receptor inhibitors (VEGFRi).

With the incorporation of new systemic therapy, namely, immune checkpoint inhibitors (ICI) with or without VEGFRi, the role and timing of cytoreductive nephrectomy (CN) are debated in patients who do not have mass-related symptoms such as ongoing hematuria or uncontrolled pain (3, 4). Nevertheless, it remains an enduring facet in the treatment landscape especially in patients where complete resection of known disease sites can be achieved. We present a case of patient with oligometastatic RCC who received upfront ICI doublet and subsequently underwent CN. We explore the role of CN in the expanding treatment landscape of metastatic ccRCC.

## Objective

We present a case of cT3aN0pM1G4 renal cell carcinoma with sarcomatoid differentiation (IMDC intermediate risk) with biopsy-proven pulmonary oligometastatic disease, in a Jehovah’s Witness man.

## Case description

**Figure 1:**
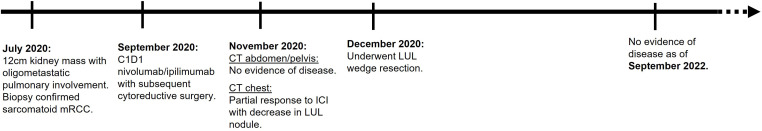
Timeline of disease.

A 62-year-old man who is a Jehovah’s Witness presented to an outside hospital in July 2020 and received antibiotics for presumed urinary tract infection (**Figure 1**). Imaging showed a large left renal centrally necrotic mass with tumor thrombosis without adenopathy, concerning for renal cell carcinoma. His past medical history included hypertension and gouty arthritis. He had no major prior surgical history. The patient was chronically on allopurinol. He drank wine socially and did not smoke or use recreational drugs. His family history includes heart disease and diabetes mellitus (parent).

The patient underwent urologic evaluation. Upfront nephrectomy was deferred due to suspicion of metastatic disease. In addition, the procedure was felt to be too high risk for perioperative bleeding which would require blood transfusions, a supportive procedure that would go against his faith. An MRI of the abdomen and pelvis revealed a 12-cm left kidney mass with extension into the left renal vein. Additionally, CT chest imaging demonstrated a 1.3-cm right lower lobe (RLL) and a 1.1-cm left upper lobe (LUL) nodule. There was no evidence of other metastatic involvement.

The patient underwent a left-lung CT-guided biopsy of the LUL nodule which showed pathological findings consistent with poorly differentiated carcinoma of renal primary. After meeting with medical oncology, he then received his two cycles of systemic therapy consisting of nivolumab and ipilimumab starting September 2020 which he tolerated well and without gouty arthritis flares.

The patient subsequently presented to our institution for a second opinion. On initial evaluation, his vital signs were within the normal range. The physical exam was non-contributory, and the patient’s performance status was excellent (ECOG 0, Karnofsky 100%). The IMDC score was 1 (intermediate, <1 year from time of diagnosis to systemic therapy) (**Table 1**).

**Table 1:** Baseline characteristics.

Initial characteristics	Values (normal range)
Time from diagnosis to systemic therapy	<1 year
ECOG/Karnofsky Performance Score	ECOG 0/KPS100
Sodium level	139 mmol/l (136–145 mmol/l)
Chloride level	102 mmol/l (98–107 mmol/l)
Creatinine level	0.80 mg/dl (0.7–1.3 mg/dl)
Calcium level	9.6 mg/dl (8.6–10.3 mg/dl)
Lactate dehydrogenase (LDH)	346 unit/l (140–271 unit/l)
Hemoglobin A1c (HbA1c)	5.8% (≤5.6%)
Aspartate aminotransferase level (AST)	22 unit/l (7–52 unit/l)
Alanine aminotransferase level (ALT)	27 unit/l (13–39 unit/l)
Hemoglobin level (Hgb)	12.6 gm/dl (12.9–16.1gm/dl)
Postoperative Hgb level	10.8 gm/dl (12.9–16.1gm/dl)
MCV (mean corpuscular volume)	83.9 fl (79.0–92.2 fl)
White blood cell count (WBC, 10^3^)	6.8 cells/µl (4.2–9.1 cells/µl)
Absolute neutrophil count (ANC, 10^3^)	4.59 cells/µl (0.67–6.4 cells/µl)
Platelet count (10^3^)	336 cells/µl (150–400 cells/µl)

At a multidisciplinary tumor board discussion, a surgical resection of his known sites of disease was felt to be feasible given oligometastatic disease status and repeat CT imaging demonstrating pulmonary nodule interval improvement following two cycles of ICI.

**Figure 2:**
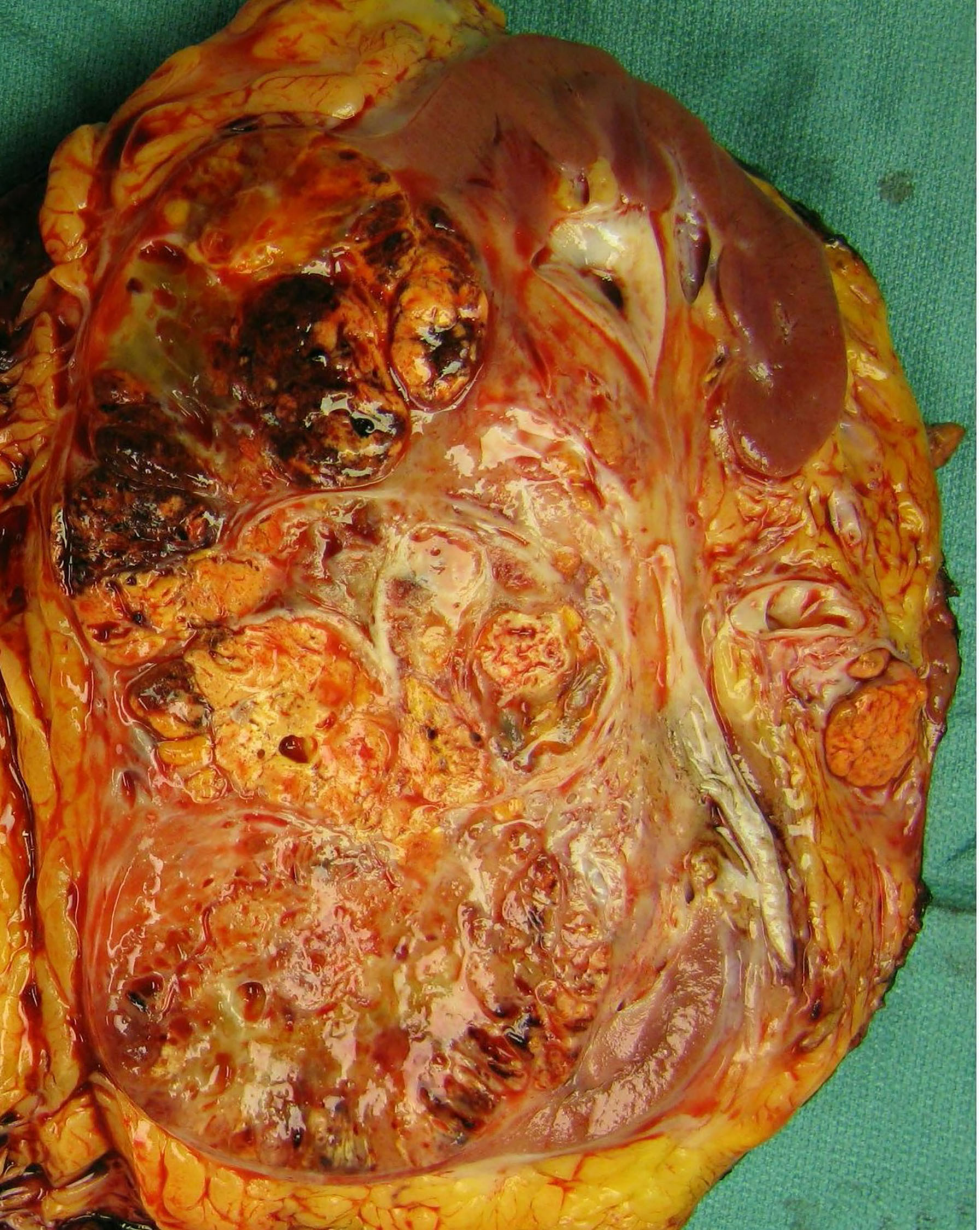
Gross examination revealed a 13.0-cm mass arising from the inferior renal pole with involvement of the interpolar region. The mass is multilobulated and variegated yellow-tan in color with areas of hyalinization and hemorrhage. It extends into the renal vein as a tumor thrombus.

The patient then underwent an open left radical nephrectomy end of September 2020, with left adrenalectomy, opting for only bloodless products as needed. There was a tumor thrombus in the left renal vein. Hemostasis was carefully maintained with Bovie electrosurgery and argon beam plasma coagulation, along with hemostatic products achieving minimal intraoperative blood loss of 150 cc. Therefore, there was no clinical need for bloodless product transfusion. The patient tolerated his procedure well; an image of the resected mass is shown in **Figure 2**. Pathology revealed a WHO nuclear grade 4 with necrosis measuring 13.0 cm in greatest dimension and invading into the renal vein (**Figure 2**). Surgical margins were negative. An incidental papillary renal cell carcinoma mixed type 1–2 measuring 3 mm in greatest dimension.

**Figure 3:**
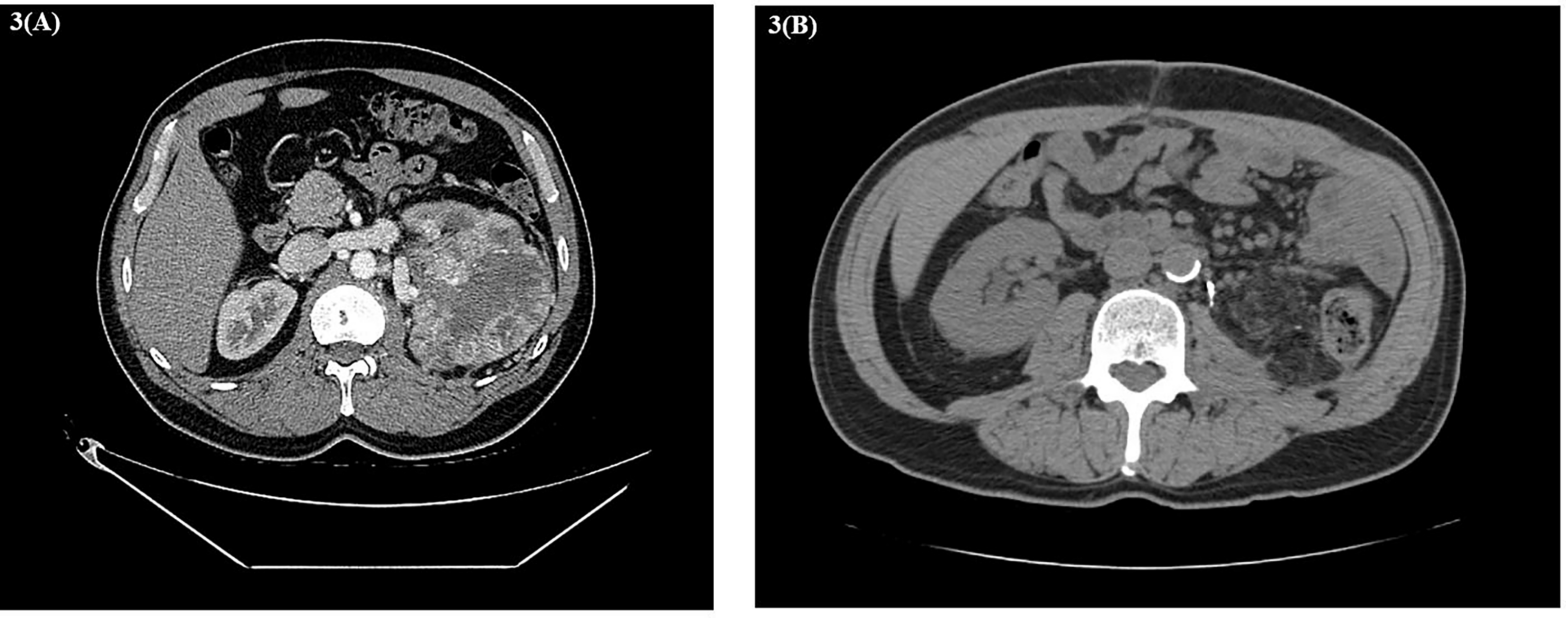
**(A)** Baseline CT abdomen and pelvis with left renal cell carcinoma. **(B)** CT abdomen 2 months after left radical nephrectomy.

**Figure 4:**
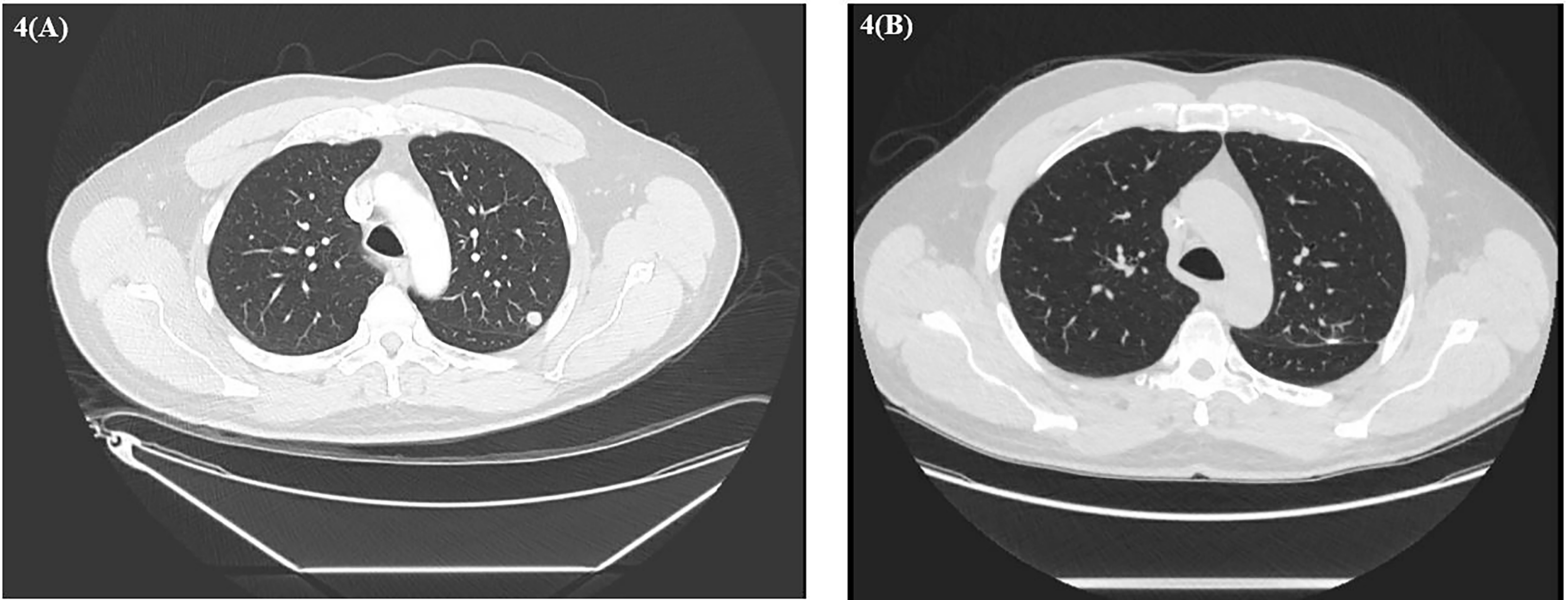
**(A)** Baseline CT chest demonstrating left upper lobe nodule. **(B)** CT chest after left thoracoscopic wedge resection.

About 6 weeks post-nephrectomy, a CT abdomen/pelvis in November 2022 revealed no evidence of disease (**Figure 3**) and the patient had recovered without major difficulties. Repeat CT chest imaging in November 2020 also demonstrated a continued decrease in the LUL pulmonary nodule and resolution of the previously noted RUL nodule. Subsequently, the patient underwent an incomplete left upper lobe wedge resection of the previously biopsy-proven metastatic LUL nodule, with pathology showing a 3-mm nodular scar and no residual malignant cells (**Figure 4**).

Given the absence of disease sites on post-ICI, post-nephrectomy, and post-wedge resection imaging, the patient was followed with active surveillance only. At the time of manuscript writing, the patient had remained for 24 months post-nephrectomy with no evidence of disease, off any systemic therapy, and without new relevant symptoms.

## Discussion

We present a case of a 62-year-old man who is a Jehovah’s Witness achieving ongoing complete response following ICI and cytoreductive surgery of oligometastatic renal cell carcinoma. The presented patient had complete surgical resection of his primary RCC tumor and complete resolution of a known metastatic pulmonary site that is attributed to nivolumab/ipilimumab therapy plus subsequent wedge resection. The case is an argument for secondary cytoreductive nephrectomy, especially in patients who could have resection of all sites of disease and who have had a disease response on initial ICI therapy. Further, our work highlights the potential for safe radical nephrectomy at tertiary centers with a high case load and where a bloodless transfusion or cell saver program exists if needed.

Several case reports highlight the use of bloodless transfusion programs to lower the risk of hemorrhage in surgical resection of vascular-rich tumors. There is a wide spectrum of blood-conserving approaches including circulatory bypass, intraoperative cell saver therapy consisting of antifibrinolytics, fibrinogen cryoprecipitate, or prothrombin concentrate (5, 6). The bloodless transfusion approach at our institution is based on informed consent and discussion of each specific product the patient would and would not be willing to receive. In this case, he was amenable to receiving cell saver, cryoprecipitate, albumin, Arista, Surgicel, and Floseal and opted against receiving fresh frozen plasma and packed red cells. Ciancio et al. presented a case series of seven patients with renal or adrenal cancer who underwent a surgical resection using techniques derived from transplantation surgery that allow for optimal intraoperative exposure and vascular control. All patients recovered well without major blood loss or perioperative complications, similar to our patient’s case. Those findings along with our case highlight the feasibility of such complex oncological surgeries without the need for blood transfusions in patients who are Jehovah’s Witnesses (7).

Two previous randomized trials cemented CN as a standard of care for patients with metastatic RCC in the cytokine era of interferon-alpha or IL-2 therapies, both of which are rarely used now and much less effective compared with the currently available systemic therapy ICI-based options. In the EORTC 30947 and SWOG 8949 trial pooled analysis, OS was significantly higher within the nephrectomy group (13.6 months) than when treated with interferon (7.8 months) (8, 9).

The role and timing of CN in the VEGFi era was once again revisited with the SURTIME and CARMENA trials. SURTIME (EORTC 30073) was a prospective trial comparing nephrectomy with sunitinib and sunitinib for three cycles with delayed cytoreductive nephrectomy (10). The two arms demonstrated an equivalent progression-free rate (PFR) at 28 weeks. OS favored deferred cytoreductive nephrectomy (32.4 months) when compared with immediate surgical intervention (15.1 months, HR 0.57, 95% CI 0.34–0.95). CARMENA (Clinical Trial to Assess the Importance of Nephrectomy) was the largest prospective, randomized, open-label, non-inferiority trial comparing patients who received sunitinib alone with those who received a nephrectomy and received adjuvant sunitinib (11). The results showed that sunitinib alone was non-inferior to nephrectomy followed by sunitinib for OS. There was no observed difference (23.6 months in sunitinib alone vs 22.7 months in adjuvant sunitinib). In the subgroup analysis, there were worsened patient outcomes in those receiving cytoreductive nephrectomy (16.6 months) vs sunitinib only [(31.2 months, HR 0.61, 95% CI 0.41–0.91)] in the setting of intermediate- or poor-risk disease. However, the trial is often criticized for having had poor accrual, having had frequent crossovers between the arms, and having been completed at a time when the treatment landscape of RCC was already shifting toward ICI-based therapies that are more effective than sunitinib (12).

Data are limited on the role and timing of CN in the current era of ICI. Proponents of the CN approach argue that the majority of patients enrolled in the IO/IO or IO/VEGF randomized clinical trials had undergone nephrectomy prior to initiation of systemic therapy (13–16). For example, 82% of patients in the Checkmate 214 of ipilimumab plus nivolumab in metastatic RCC had a prior nephrectomy. Further, CN offers the theoretical benefit of reducing the tumor burden of the primary, which could otherwise serve as a highly vascular and immunogenic sink for the administered systemic therapy drugs. Opponents to the CN approach shed concerns on the retrospective or *post-hoc* nature of data in favor of CN and the potential for major complications from a nephrectomy. In addition, *post-hoc* data have mixed results. For instance, a *post-hoc* analysis of the CLEAR trial showed that patients with mRCC who had an intact primary (a minority of patients in the trial) had a relatively improved clinical benefit (OS, PFS, ORR) from lenvatinib and pembrolizumab vs sunitinib that is similar to the one observed in the overall trial population (17).

A balanced viewpoint favors multidisciplinary collaboration as key to appropriate patient selection. Expert guidelines continue to support CN for patients with a favorable-intermediate IMDC risk score, low-volume or oligometastatic disease, and absence of brain metastases. In patients with intermediate- or poor-risk disease, CN can still be considered in patients with a positive response to systemic therapy, especially when all disease sites can be resected. A contemporary study by Esdaille et al. consisting of 937 patients across five centers demonstrated a 10% major complication rate with 1% mortality within 30 days after CN. Systemic therapy prior to CN was not significantly associated with risk of complications or mortality, supporting the approach followed in our presented case (18). Another multicenter study assessed overall survival in 1,163 patients across five different centers who underwent upfront CN. There were 79% treated without neoadjuvant systemic therapies. Most patients were either intermediate or poor risk on preoperative IMDC stratification. Of the 245 patients stratified into poor-risk IMDC, OS was not associated with comorbidity or age. Median overall survival was 21.8 months in poor-risk patients significantly exceeding that in the cohorts examined in CARMENA and SURTIME (19).

Prospective clarification is ongoing for the role of deferred CN in the ICI era. Phase III trials include the NORDIC-SUN (NCT03977571) trial of nivolumab/ipilimumab with or without CN and the PROBE (NCT04510597) of standard-of-care first-line therapy with or without CN. Nivolumab and cabozantinib with or without CN is also being evaluated in the Cyto-KIK trial (NCT04322955). ICI without or without stereotactic body radiation therapy (SBRT) is also explored as treatment of the primary in the CYTOSHRINK (NCT04090710, phase II) trial.

## Conclusion

Cytoreductive nephrectomy is an important and often debated facet in treatment of metastatic renal cell carcinoma. With a rapidly expanding treatment landscape of ICI/ICI and ICI/VEGF, its definitive role and timing are less well defined. We hope more prospective and real-world data will benefit our understanding regarding the timing and role of ICI-based therapy and CN in patients with metastatic RCC.

## Data availability statement

The original contributions presented in the study are included in the article/Supplementary Material. Further inquiries can be directed to the corresponding author.

## Ethics statement

Written informed consent was obtained from the individual(s) for the publication of any potentially identifiable images or data included in this article.

## Author contributions

BN and VM have contributed equally to this work and share senior authorship. All authors contributed to the article and approved the submitted version.

## Conflict of interest

BN has acted as a paid member of the advisory board of Exelis and paid participant in a case discussion for IntrinsiQ Specialty Solutions—AmerisourceBergen. MB has acted as a paid consultant for and/or as a member of the advisory boards of Exelixis, Bayer, BMS, Eisai, Pfizer, AstraZeneca, Janssen, Calithera Biosciences, Genomic Health, Nektar, EMD Serono, SeaGen, and Sanofi and has received grants to his institution from Merck, Xencor, Bayer, Bristol-Myers Squibb, Genentech/Roche, SeaGen, Incyte, Nektar, AstraZeneca, Tricon Pharmaceuticals, Genome & Company, AAA, Peloton Therapeutics, and Pfizer for work performed as outside of the current study.

The remaining authors declare that the research was conducted in the absence of any commercial or financial relationships that could be construed as a potential conflict of interest.

## Publisher’s note

All claims expressed in this article are solely those of the authors and do not necessarily represent those of their affiliated organizations, or those of the publisher, the editors and the reviewers. Any product that may be evaluated in this article, or claim that may be made by its manufacturer, is not guaranteed or endorsed by the publisher.
